# Discovering antimalarials with a differential mode of action

**DOI:** 10.1186/1475-2875-13-S1-P79

**Published:** 2014-09-22

**Authors:** Laura Sanz, Maria Jose Lafuente, Sara Prats, Fernando Neria, María Roncalés, Cristina de Cózar, Ane Rodríguez, María Gómez, Francisco Javier Gamo

**Affiliations:** 1 Malaria DPU, Diseases of the Developing World (DDW), GlaxoSmithKline, Tres Cantos Madrid, 28760, Spain

## 

Malaria is a global health problem that causes significant mortality and morbidity, with more than 1 million deaths per year caused by *P. falciparum*. Most antimalarial drugs face decreased efficacy due to the emergence of resistant parasites. Therefore, the efforts to improve current treatments are focused on the identification of new antimalarial drugs displaying novel mechanisms of action.

To increase the probability of success to identify compounds active against all relevant parasite drug targets, phenotypic assays have been used to screen large compound libraries against *P. falciparum* blood stages. At GSK, 2M compounds were screened and 13500 confirmed hits (TCAMS-Tres Cantos Antimalarial Set) were selected which inhibited *P. falciparum* growth with IC_50_s below 2 μM.

In order to select chemotypes with a presumptive novel mechanism of action, an early profiling is carried out which involves several phases:

1. In the first phase, compounds are tested against a wide panel of *P. falciparum* strains with well characterized genetic linkage to drug resistance to establish which series had no cross-resistance with already known antimalarial compounds. Panel includes laboratory strains as well as clinical isolates.

2. In a second phase, compounds are tested using functional assays to identify those molecules interfering with parasite pathways that are targets of already known antimalarials (electron transport, folate and isoprenoid). Both mitochondrial electron transport and pyrimidine biosynthesis inhibitors are identified using transgenic parasites overexpressing *Saccharomyces cerevisiae* DHODH. Addition of proguanil to cultures restores cytochrome bc1 blockage but not DHODH inhibition. Antifolates are identified by supplementing the growth medium with folic acid and, similarly, inhibitors of the isoprenoid biosynthesis pathway by adding geranyl geranyol or isopentenyl pyrophosphate, which shifts the IC_50_ of inhibitors of these pathways.

3. Finally compounds are subjected to hematin polymerization assays to establish the interference with this essential pharmacological target of malaria parasites.

New scaffolds with novel modes of action can be selected using these approaches avoiding the interference with pathways which are known to develop resistance. This strategy has already been succesful and our current antimalarial pipeline is based on scaffolds displaying new mechanisms of action. Chemoproteomics and chemical genomics efforts are being used to decipher new MoA.

